# Self-assessment of residents in breaking bad news; skills and barriers

**DOI:** 10.1186/s12909-023-04720-4

**Published:** 2023-10-06

**Authors:** Maryam Mansoursamaei, Atefeh Ghanbari Jolfaei, Mehdi Zandi, Ali Mansoursamaei, Razieh Salehian

**Affiliations:** 1 Student Research Committee, School of Medicine, Shahid Beheshti University of Medical Sciences, 19857-17443 Tehran, Iran; 2 Minimally Invasive Surgery Research Center, Department of Psychiatry, School of Medicine, Iran University of Medical Sciences, 14456-13131 Tehran, Iran; 3 Student Research Committee, School of Medicine, Iran University of Medical Sciences, 14456-13131 Tehran, Iran; 4 Student Research Committee, School of Medicine, Shahroud University of Medical Science, 36147-73943 Shahroud, Iran; 5 Mental Health Research Center, Department of Psychiatry, School of Medicine, Psychosocial Health Research Institute, Rasoul-e-Akram Hospital, Iran University of Medical Sciences, 14456-13131 Tehran, Iran

**Keywords:** Breaking bad news, Giving bad news, Delivering bad news, Physicians, Residents, Barriers

## Abstract

**Introduction:**

Breaking bad news (BBN) is inevitable in medicine and is one of the most important and difficult professional tasks of physicians. The main aims of this study are to evaluate residents’ practice of BBN and identify perceived barriers to its implementation.

**Methods:**

In this cross-sectional study in 2021, 240 residents from medical, surgical, and emergency medicine departments completed the demographic questionnaire, the Persian SPIKES questionnaire (P-SPIKES), and the researchers-made questionnaire of Barriers to Breaking Bad News (BBBN). In addition, they were asked about their previous experience, previous training, and their perceived level of competence in BBN.

**Results:**

46.5% and 36.84% of residents rated their perceived competence in BBN and managing the patient’s emotions during BBN as good or very good, respectively. The most difficult aspects of BBN for residents were expressing upsetting information (78.1%) and not disappointing the patient while being honest (58.3%). The mean and standard deviation of the score of the P- SPIKES was 55.92 ± 6.84. The most common SPIKES item was not giving bad news by phone (98.9%). The SPIKES total score was only related to age (positive relationship). The most commonly reported barriers to BBN were concerns about controlling the patient’s emotions (61%) and the aggressiveness of the patient or companions (52.6%). A significant proportion of participants identified lack of training (28%) and insufficient skills (21.9%) as significant barriers to BBN.

**Conclusions:**

The skill of residents in BBN is insufficient in some aspects and points to the need for BBN training courses during residency. BBN is difficult for residents in some aspects and residents may perceive barriers. To overcome the existing barriers and increase residents’ confidence in BBN, strategies such as incorporating BBN training into residency educational curricula and communication skills are recommended.

## Introduction

Bad news is defined as any information that has a negative and significant effect on an individual’s outlook on her or his future [1]. Bad news can include information related to a chronic disease, a life-altering illness, or an injury leading to significant change [2]. Patients dislike the way doctors indicate and label negative news as “unfortunate or bad” [3]. They prefer to view the news as something to process and deal with under the direction of their doctor [3]. Therefore, using “serious news” is recommended instead of bad news (4). Many patients seek out trustworthy information before making important decisions. However, those who find it too serious may exhibit behaviors like denial, shunning, or downplaying the significance of the knowledge, while still receiving treatment (5). It is unproven that getting serious medical information will invariably result in psychological harm (5). Therefore, even if doctors suspect it could harm the patient, they are not permitted to conceal medical information (6). Most patients prefer to know their diagnosis, but the amount of information they seek out varies depending on culture, level of education, age, and gender (2, 7–9). Younger patients, females, and those with high education levels prefer more comprehensive and detailed information (2). Cultural norms and ethnicity also have an impact on the amount of desired information. According to one study, Korean Americans and Mexican Americans prefer a family-centered medical decision model, whereas African Americans and European Americans prefer a model with greater individual patient autonomy (2). Given these notifications, physicians must notice and inquire about patients’ preferences before giving serious news [2].

There have been some recommendations for the giving bad news over the years that are primarily based on expert opinion rather than empirical evidence [10]. These universal models have been proposed and utilized effectively to guide and enhance the delivery of bad news among physicians. One of the most popular guidelines for breaking bad news (BBN), especially for cancer patients, is the SPIKES protocol [1].

The SPIKES protocol is an acronym that refers to six steps recommended for BBN: (S) setting up the interview; (P) assessing the patient’s perception; (I) obtaining the patient’s invitation; (K) giving knowledge to the patient; (E) addressing the patient’s emotions with empathic responses; and (S) strategy and summary (1). The first step “S”, or setting up, refers to the preparation of the environment, preferably in a private, reserved, and welcoming place. The second phase, “P,“ or perception, relates to determining whether the individual who received the news is aware of their own condition or disease by asking open-ended questions to assess the patient’s knowledge, expectations, and readiness to take the information. The third phase “I”, invitation, entails determining to whom and to what extent the patient wishes to disclose the news. The fourth stage “K” is knowledge, in which information is presented with a caring and honest demeanor, according to the patient’s needs and desires. Step “E”, Emotions, entails observing and listening to the patient, identifying the patient’s concerns, emotions, and reactions, and managing the patient’s emotions by employing identifying, acknowledging, and validating statements. The last step “S”, summary and strategy, is an important step for summarizing everything that has been delivered and checking whether the patient has understood it. It also provides an opportunity to develop a strategy in cooperation with the patient and identify the patient’s support resources [1, 5].

Aside from these steps, mental preparation is essential for those who are tasked with delivering serious news. They must review the patient’s condition, previous treatments, results, and scripts, anticipate the patient’s questions about the prognosis and treatment failure, and prepare to manage the patient’s emotions (11). It is also beneficial to note any previous discussions with other healthcare workers to gain an understanding of the patient’s prior knowledge and expectations [11]. As a result, the SPIKES was modified in 2005 to the PSPIKES. Users of this model reported more confidence in their ability to discuss unfavorable medical information with their patients [11].

Physicians must be able to deliver bad news [12]. BBN is one of the most important duties of physicians and medical staff, despite its importance, barriers to BBN have been identified. According to research, time constraints, language differences, personal fears, the illiteracy of the patients, crowded wards with no privacy, and a lack of training are all significant barriers to BBN [13].

Most undergraduate and graduate medical programs do not typically provide specialized training in BBN [14]. There is relatively little information on how residents deliver bad news in recent years. Previous studies had limitations such as small sample sizes, different study samples, and being limited to only a few specialties. Some of these limitations are addressed in this study.

The aims of the current study were to survey the skill of residents in medical, surgical, and emergency medicine departments regarding BBN based on the SPIKES protocol and to identify perceived obstacles to implementing it. We also looked at doctors’ demographic characteristics, their training in communication skills, and their experience with serious news, all of which can influence how physicians deliver bad news. Furthermore, we asked general questions about BBN, such as how often they deliver bad news and the extent of their need to receive additional academic training. The findings of the study can provide information about the current practice of residents in delivering bad news and increase their proficiency by assisting policymakers in developing the necessary strategies to overcome perceived obstacles.

## Methods

This cross-sectional study was conducted in 2021 at two general medical hospitals affiliated with Iran University of Medical Science. Participants included residents from medical, surgical, and emergency medicine departments who agreed to fill out the questionnaires and participation was voluntary and anonymous. All residents who agreed to complete the questionnaires were included in this study, but the residents who did not have a strong clinical performance in their career (radiologists and pathologists) and those whose responses to questionnaires were incomplete were excluded. At first, demographic data and some questions related to residents’ opinions about delivering bad news, their previous experiences of receiving or delivering bad news, and their prior training in BBN were assessed. Then, the participants were asked to complete the SPIKES and the barriers to BBN questionnaire.

### Material

### The Persian questionnaire of SPIKES (P-SPIKES)

P-SPIKES showed good reliability and validity [15]. This questionnaire includes 16 questions and two psychological and environmental domains. The items are scored on a 5-point Likert scale (always, often, sometimes, rarely, and never) with minimum and maximum total scores of 16 and 80, respectively; higher scores show the proficiency of the physician. Each of the domains has 8 items with a minimum score of 8 and a maximum score of 40. In the majority of questions, always and often were considered the favored option; in a few questions, rarely and never were preferred [15, 16]. Also, some questions regarding the preparation step were added to the questionnaire.

### Barriers to Breaking Bad News Questionnaire (BBBN)

We used the BBBN to identify the barriers to delivering bad news. To provide the BBBN, an initial set of items was prepared by reviewing the literature and consulting a multidisciplinary panel of experts affiliated with ***(comprising one cardiologist, one neurologist, one psychiatrist, one emergency medicine specialist, one oncologist, one internal medicine specialist, and four surgeons in different specialties). In the quantitative assessment of the content, the experts rated the items of the questionnaire on a 5-point ordinal scale to determine the potential significance of the various barriers using the formula “Impact Score = Frequency (%) ×Importance”, which frequency (%) represents how often they perceived the item as a barrier to BBN and importance shows a subjective measure of how the significance of the item is on a scale of 1 to 5 (importance = 5). Based on the minimum acceptable score of 1.5, 14 items were selected and included in the initial draft of the questionnaire [17].

To determine the face and content validity of the questionnaire, the same experts from various specialties were asked to provide corrective opinions on the 14 items. In the qualitative assessment, the experts were asked to offer their opinions about each item in terms of proper words, grammar, understandability of the items, and appropriate time for completion of the questionnaires to modify the items according to their feedback. The content validity index (CVI) was used to determine to the extent each item was in terms of clarity, simplicity, and relevancy on a 4-point ordinal scale (from 1 = the lowest to 4 = the highest). A CVI > 0.79 was considered acceptable [18]. The content validity rate (CVR) used to determine whether an item was necessary in the questionnaire on a 3-point ordinal scale. According to the Lawshe method given a panel size of ten experts, a CVR > 0.62 was considered acceptable [19].

Finally, 25 residents completed the questionnaire twice with an interval of 10–14 days to determine the temporal reliability of the BBBN using the test-retest method. The CVI, CVR, and temporal reliability of the questionnaire were calculated to be 0.94, 0.85, and 0.81, respectively. The final questionnaire consisted of 11 items focusing on 10 barriers and an open-ended item that inquired about additional items. Based on the acceptable results, this questionnaire was used to collect the information required for this research.

### Sample size

The ratio estimation formula was used and an initial sample size of 216 people was calculated. Considering 10% of the non-response rate, 240 eligible residents participated in the study by convenience sampling method as the final sample size.

### Ethics

The study protocol was approved by the Research Ethics Committee of ***.

### Data analysis

The data were analyzed using SPSS version 22. First, descriptive statistics analyses including frequencies, percentages, means, and standard deviations, were used to measure characteristics of the participants and their experiences with bad news. The distribution of the normality of the P-Spikes total score was examined using the Kolmogorov–Smirnov test. The means of P-Spikes domains were compared using independent t-test. The association between the mean of P-Spikes total score and categorical variables determined using independent sample t-test and one-way ANOVA (respectively for two groups and more than two groups of the variable). Pearson’s correlation test was used for association of P-Spikes total score and age. P-value ≤ 0.05 was considered significant.

## Results

After excluding the uncompleted questionnaires of the 240 participants, the data of 228 first- to fourth-year residents in medical, surgical, and emergency medicine departments were included in this study. Table 1 shows the participants’ demographic data and attitudes toward delivering bad news.

**Table 1:** **Sociodemographic characteristic of the residents and their experiences of bad news**

Quantitative variables	(Mean ± SD)
**Age** (years)	30 ± 3.3
**Average time for delivering bad news** (minutes)	10.93 ± 6.8
**Qualitative variables**	**No. (%)**
**Gender**	
Male	130 (57%)
Female	98 (43%)
**Marital status**	
Married	89 (%39)
Divorced	8 (%3.5)
Single	131 (57.5%)
**Years of medical experience**	
<5	199 (87.3%)
5–10	20 (8.8%)
>10	9 (3.9%)
**Training department**	
Emergency Medicine	28(12.3%)
Surgical	90 (39.5%)
Medical	110 (48.2%)
**The necessity for giving bad news**	
Shouldn’t be given	25 (11%)
Isn’t necessary	4 (1.8%)
Should be given	199 (87.3%)
**Experience of receiving bad news**	
Yes	189 (82.9%)
No	39 (17.1%)
**Experience of giving bad news during residency**	
Yes	218 (95.6%)
No	10 (4.4%)
**Frequency of giving bad news in the past month**	
0 to 5 times	156 (68.4%)
5 to 9 times,	50 (21.9%)
10 to 14 times	13 (5.7%)
15 times or more	9 (3.9%)
**Training to give bad news**	
Formal training	103 (45.2%)
Clinical training	18 (7.9%)
Both	26 (11.4%)
Neither	81 (35.5%)
**Training in dealing with the patient’s emotions**	
Formal training	69 (30.3%)
clinical training	14 (6.1%)
Both	15 (6.6%)
Neither	130 (57%)
**The most difficult part of giving bad news**	
Expressing upsetting information	178 (78.1%)
Not disappointing the patient while being honest	133 (58.3%)
Addressing the patient’s emotions	102 (44.7%)
Involving the patient’s family and relatives	35 (15.4%)
Participating the patient/family in decision-making	27 (11.8%)
**Perceived ability to deliver bad news**	
Very good	12 (5.3%)
Good	94 (41.2%)
Average	98 (43.0%)
Weak	23 (10.1%)
Very weak	1 (0.4%)
**Perceived ability to face the patient’s emotions**	
Very good	13 (5.7%)
Good	71 (31.1%)
Average	111 (48.7%)
Weak	28 (12.3%)
Very weak	5 (2.2%)
**The most suitable candidate for giving bad news**	
Doctor	217 (95.2%)
Patient’s accompaniments	5 (2.2%)
Nurses	2 (0.9%)
Department Secretary	2 (0.9%)
I don’t know	2 (0.9%)

SD: standard deviation; No. (%): number (percentage)

31.6% of residents reported delivering bad news to their patients more than five times in the previous month and reported an average of 10.93 ± 6.8 min for each patient. On a 5-point scale, 46.5% and 36.84% of residents rated their perceived competence in BBN and managing the patient’s emotions during BBN as good or very good, respectively. The most difficult aspects of giving bad news were expressing upsetting information (i.e., new serious medical information, relapse of disease, death, or permanent loss of an organ or organ function) (78.1%), and not disappointing the patient while being honest (58.3%).

**Fig. 1:**
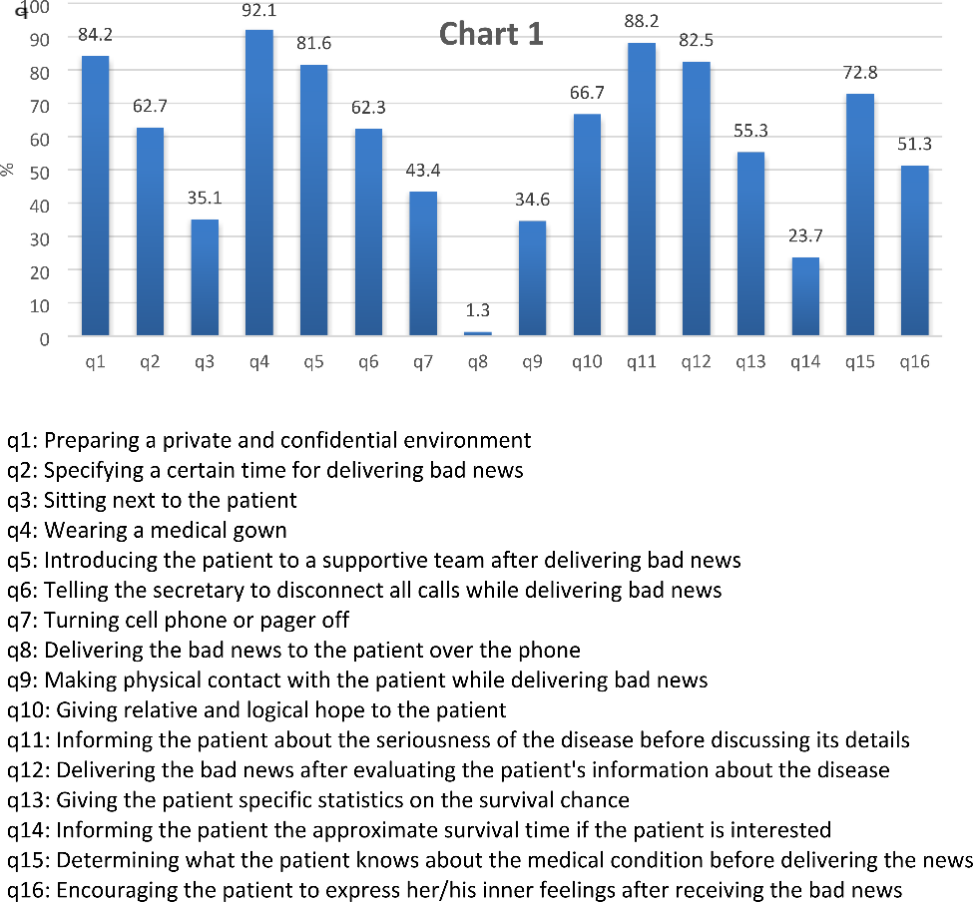
Skills of residents for giving bad news

Figure 1 demonstrates the percentage of different skills used by residents for delivering bad news.

The cumulative percentage of always and often responses to each P-SPIKES item is shown.

According to the findings, among the SPIKES skills, not giving the bad news over the phone (98.9%) and wearing a medical gown while delivering the bad news (92.1%) were most common, while informing the patient of the approximate survival time if she/he is interested (23.7%), making physical contact with the patient (34.6%), and sitting next to the patient (35.1%) were the least frequent when delivering the bad news. Regarding resident characteristics, only gender showed an association with the items of P-SPIKES; male residents were more likely to arrange for privacy (p = 0.007) and to inform the patient of the approximate survival time (p = 0.012), whereas female residents were more likely to wear a medical gown (p = 0.001) and to break the bad news in person (p = 0.006). There was no significant difference between the residents from different departments (P > 0.05).

In response to the two questions from the preparation stage, which were not included in the SPIKES questionnaire, but we inquire of residents, the majority of participants (73%) stated that they review the medical history and mentally rehearse giving bad news. Also, 83% stated that they prepare themselves to face and address the patient’s emotions before BBN.

The P-SPIKES total score had a mean and standard deviation of 55.92 ± 6.84 (Table 2). No statistically significant distinction was observed between the two domains of delivering bad news (P = 0.072).

**Table 2:** P-Spikes: Total score and the score of its domains

	N	Minimum	Maximum	Mean	SD
Environmental	228	16.00	38.00	26.47	4.04
Psychological	228	14.00	34.00	25.99	3.32
P-SPIKES total score	228	33.00	73.00	55.92	6.84

P-Spikes: The Persian questionnaire of SPIKES; SD: standard deviation

Table 3 shows the association between the variables and the SPIKES total score. The skill of residents to BBN was only related to age (p = 0.014). Older residents were significantly more likely to arrange for privacy (p = 0.041), ensure adequate time (p = 0.004), sit next to the patient (p = 0.027), assess the patient’s perception of her/his medical condition before BBN (p = 0.023), try to determine how much the patient wants to know (p = 0.036), and introduce the patient to a supportive team after BBN (p = 0.031).

**Table 3:** Association of residents’ characteristics and their experience of bad news with their skills in BBN

Variable	(Mean ± SD)	p-value	
Gender*		p > 0.05	
Male	56.3 ± 7.1		
Female	55.4 ± 6.4		
Marital status**		p > 0.05	
Single	55.9 ± 7.2		
Married	55.9 ± 6.4		
Divorced	54.6 ± 3.7		
Training department**		p > 0.05	
Emergency Medicine	56.7 ± 5.7		
Medical	55.9 ± 6.4		
Surgical	55.6 ± 7.6		
Years of work experience**		p > 0.05	
<5	55.8 ± 6.9		
5–10	56.8 ± 5.9		
>10	56.5 ± 5.7		
Experience with receiving bad news*		p > 0.05	
No experience	56.5 ± 7.5		
Prior experience	55.7 ± 6.9		
Training in delivering bad news**		p > 0.05	
No prior training	55.1 ± 6.2		
Theoretical training	56.5 ± 7.3		
Clinical training	55.6 ± 5.8		
Both	56.2 ± 7.2		
Training in dealing with the patient’s emotions**		p > 0.05	
No prior training	54.9 ± 6.3		
Theoretical training	57.1 ± 7.4		
Clinical training	56.0 ± 3.8		
Both	58.2 ± 8.7		
Age***	(R: 0.17)	p = 0.014	

*Independent t test, **one way ANOVA test, and ***Pearson’s correlation analysis was used for association of variables and P-Spikes total score. p < 0.05 was considered significant. BBN: breaking bad news; Mean ± SD: Mean ± standard deviation; R = Pearson’s correlation coefficient

Regarding the barriers to BBN that residents introduced, the results of BBBN showed that concerns about controlling the patient’s emotions (61%), the aggressiveness of the patient or the companions (52.6%), and the reluctance of the patient’s companions to give bad news to the patient (39.9%) were among the most prevalent barriers to giving bad news among medical residents (Table 4).

**Table 4:** Barriers to breaking bad news perceived by residents (BBBN Responses)

Barriers		N	%
B1	Concern about controlling own emotion	23	10
B2	Concern about controlling patient’ emotion	139	61
B3	Concern about the aggressiveness of patients or companions	120	52.6
B4	Insufficient skill and experience in giving bad news	50	21.9
B5	Lack of prior training in giving bad news	64	28
B6	Insistence of the companions not to implement the bad news	91	39.9
B7	Concern about breaking the therapeutic relationship or abandoning treatment	43	18.9
B8	Not having enough time	43	18.9
B9	Lack of suitable place	81	35.5
B10	Difficulty communicating verbally due to the patient’s condition (such as patient illiteracy, hearing loss, speech problem) or having a different language or accent	37	16.2

BBBN: Barriers to Breaking Bad News Questionnaire; No: number; (%): percentage

## Discussion

The current research aimed to evaluate residents’ skills in BBN and to identify perceived barriers to BBN. In the present study, about one-third of respondents reported frequently breaking bad news (more than five times in the last month). The study found that residents don’t perform well in some steps of BBN, particularly empathy and nonverbal delivery (allocating time, sitting down, making physical contact), but they perform better in preparation step and verbal aspects of delivering bad news, such as correctly applying the steps of invitation and knowledge. The vast majority of residents (87.3%) agreed that bad news should be delivered to the patient and identified the physician as the best person to deliver bad news (95.2%). Despite this, more than half rated their perceived ability to deliver bad news as not good, and approximately two-thirds rated their ability to manage patients’ emotions as moderate or low. The principles of informed consent, patient autonomy, and case law have established clear ethical and legal obligations to provide patients with as much information about their illness and treatment as they desire [20].

The lack of formal training in BBN as a basic clinical task may be one of the factors contributing to low self-confidence and feeling difficulty to do BBN (21). It is worth noting that education will not facilitate communication in BBN unless it is combined with training. Training will allow clinicians to overcome the stress of BBN and develop confidence(22–24). In the current study, a significant proportion of residents reported having no theoretical or clinical training in either BBN (35.5%) or dealing with the patient’s emotions (57%). These findings are consistent with previous studies in our country, though it seems formal training for BBN has improved in recent years [25–27]. The findings were also in line with research from other countries. A cross-sectional survey that targeted all healthcare providers of the intensive care units of 40 countries revealed that only one-third had received formal training [28]. This survey found that younger healthcare workers and those with fewer years of work experience had been trained less [28]. The current study showed that the ability to BBN (as measured by the SPIKES score) was only associated with age and has not shown a significant relationship with years of professional experience and training specialty (medical, surgical, and emergency medicine). Differences in the SPIKES scores may be related to factors such as the age, gender, and cultural background of the residents [29]. Since awareness of the need for training in BBN has increased in health care over time, more residency programs have incorporated BBN training into their curriculum. However, the ratio of formally trained healthcare providers is not proportional to the anticipated need, highlighting the global need to develop BBN educational courses [28].

Another important finding of this study was that there was no significant difference in performance between residents with and without prior training, suggesting that the current training programs are ineffective and should be modified. Implementing a strategy in which residents adhere to a predetermined protocol is also critical. Lectures, small-group discussions, role-playing with peers and standardized patients (SPs), and learning in the context of patient care are all potential strategies for providing education in the delivery of bad news [21].

According to another finding of the study, the most difficult aspects of giving bad news were conveying upsetting information to the patient, not disappointing the patient while being honest, and addressing the patient’s emotions. These difficulties can be greatly facilitated by using communication skills and several strategies, such as exploring the patient’s knowledge, expectations, and hopes (5). BBN is a complex communication task, of which the verbal component is only one. So, it requires other skills, such as responding to patients’ emotional reactions, patient involvement in decision-making, coping with the stress arising from patient expectations, the participation of multiple family members, and the manner of how to give hope in an unfavorable conditions [5]. BBN can be facilitated by understanding the process, using a step-by-step approach, and applying well-established communication and counseling principles [30].

BBN has been shown to be necessary and vital in reducing the traumatic effects of bad news on patients’ illness perceptions, disease coping, and life expectancy, and could encourage the patient to engage actively in difficult decisions.(31–33) However, the personal and institutional barriers make it difficult for residents to apply guidelines. Barriers, such as the aggressiveness of the patient or companions and the reluctance of the patient’s companions to BBN to their patient, were defined as more common in the current study than in other studies [34]. Bad news delivery can be influenced by culture-bound attitudes, religious values, and medical traditions [35]. In comparison to the United States [36], research in Brazil [37], Sudan [38], Saudi Arabia [39], and Korea [40] revealed a higher rate of bad news transmission to the family rather than directly to the patients, and families are heavily involved in the patient’s decisions. In this study, the most common barrier to giving bad news was concern about controlling the patient’s emotions. Furthermore, a significant proportion of participants identified a lack of training or insufficient skills as barriers to BBN. Empathy is one of the best methods to help patients feel less alone and validate their thoughts and feelings [5]. Communication skills training and the development of a therapeutic relationship positively impact patients and their relatives [30]. Although patients value knowledge and professional guidance, focusing solely on facts and ignoring the patient’s emotional requirements results in less positive responses than prioritizing the patient’s emotional needs [41]. Of importance, medical education, particularly during internship and residency, has the potential to suppress empathy and replace communication with techniques and procedures [42]. The problem is partly due to the tendency of medical students to prioritize technical proficiency over the significance of communication skills [26]. As a result, communication proficiency among medical students tends to decline as they progress through their academic programs [42]. This decline contrasts with the current model of patient-centered communication, as patients desire better communication with their physicians [42]. Patients prefer to receive serious news in person and with the physician’s undivided attention, but they also want to have faith in the physician’s competence [2, 43]. A good physician-patient relationship has the potential to help with emotional adjustment and increase patient satisfaction and compliance [42]. In recent years, universities and other institutions have made significant efforts in the form of courses, forums, and available printed materials to help physicians improve their abilities and knowledge in this challenging area [44].

There are some limitations to this study. First, the data were obtained through a self-reporting cross-sectional study, which may not have accurately represented the residents’ clinical performance. Second, the study was limited to residents practicing in two general hospitals. However, residents from different disciplines participated in this study. Finally, this study did not assess whether the specific guidelines affected patients’ satisfaction or outcomes. Despite these limitations, this study had numerous strengths; residents of different disciplines in three general groups of medical, surgical, and emergency medicine participated in this study, so this study can provide a better and broader description of the conditions of BBN in different departments of the hospital. Also, the results indicated that along with theoretical and practical training, it is better to review the current training programs and monitor the implementation of protocols and guidelines more carefully. In addition, this study reported barriers to BBN that were under-reported in previous research that should be addressed. Additional research is needed in the areas of objective assessments by patients or doctors of how physicians deliver bad news, whether published guidelines on delivering bad news accurately reflect patients’ expressed needs and preferences, and whether delivering bad news in accordance with current guidelines affects patient satisfaction and clinical outcomes.

## Conclusions

Despite the importance of BBN in medicine, the obtained results revealed that about one-third of residents treating patients have never received any training on BBN and the skill of residents in BBN is not enough in some aspects, even among trained residents. Residents perceived the difficulty of BBN in some aspects and their incompetence in this field. They identified barriers to BBN that may be alleviated using several strategies such as the incorporation of BBN training and communication skill training courses in the educational curricula of residency. Also, physical modification of hospital departments to provide a suitable environment for BBN is important.

## Data Availability

The datasets used and/or analyzed during the current study available from the corresponding author on reasonable request.
